# *TRAF1/C5* but Not *PTPRC* Variants Are Potential Predictors of Rheumatoid Arthritis Response to Anti-Tumor Necrosis Factor Therapy

**DOI:** 10.1155/2015/490295

**Published:** 2015-03-05

**Authors:** Helena Canhão, Ana Maria Rodrigues, Maria José Santos, Diana Carmona-Fernandes, Bruno F. Bettencourt, Jing Cui, Fabiana L. Rocha, José Canas Silva, Joaquim Polido-Pereira, José Alberto Pereira Silva, José António Costa, Domingos Araujo, Cândida Silva, Helena Santos, Cátia Duarte, Rafael Cáliz, Ileana Filipescu, Fernando Pimentel-Santos, Jaime Branco, Juan Sainz, Robert M. Plenge, Daniel H. Solomon, Jácome Bruges-Armas, José António P. Da Silva, João Eurico Fonseca, Elizabeth W. Karlson

**Affiliations:** ^1^Rheumatology Research Unit, Instituto de Medicina Molecular, Edifício Egas Moniz, Faculdade de Medicina da Universidade de Lisboa, Avenida Egas Moniz, 1649-028 Lisbon, Portugal; ^2^Rheumatology Department, Hospital de Santa Maria (CHLN), Lisbon Academic Medical Centre, 1649-035 Lisbon, Portugal; ^3^Division of Rheumatology, Allergy and Immunology, Section of Clinical Sciences, Brigham and Women's Hospital, Boston, MA 02115, USA; ^4^Rheumatology Department, Hospital Garcia de Orta, 2805-267 Almada, Portugal; ^5^SEEBMO, Hospital de Santo Espirito da Ilha Terceira, Azores, 9700-049 Angra do Heroísmo, Portugal; ^6^Genetics & Arthritis Research Group (GARG), Institute for Molecular and Cell Biology (IBMC), 4150-180 Oporto, Portugal; ^7^Rheumatology Department, Conde de Bertiandos Hospital (ULSAM), 4990-041 Ponte de Lima, Portugal; ^8^Instituto Português de Reumatologia, Lisbon, Portugal; ^9^Rheumatology Department, Centro Hospitalar da Universidade de Coimbra, 3000-076 Coimbra, Portugal; ^10^Rheumatology Department, Virgen de las Nieves University Hospital, Granada, Spain; ^11^Rheumatology Department, University of Medicine and Pharmacy “Iuliu Hatieganu”, Cluj-Napoca, Romania; ^12^CEDOC, Faculdade de Ciências Médicas, Universidade Nova de Lisboa, Lisbon, Portugal; ^13^Rheumatology Department, Hospital de Egas Moniz (CHLO), 1349-019 Lisbon, Portugal; ^14^Genomic Oncology Area, GENYO Centre for Genomics and Oncological Research, Pfizer/University of Granada/Andalusian Regional Government, PTS Granada, Granada, Spain; ^15^The Broad Institute of Harvard and MIT, Cambridge, MA, USA; ^16^Division of Pharmacoepidemiology, Brigham and Women's Hospital, Boston, MA, USA

## Abstract

*Background*. The aim of our work was to replicate, in a Southern European population, the association reported in Northern populations between *PTPRC* locus and response to anti-tumor necrosis factor (anti-TNF) treatment in rheumatoid arthritis (RA). We also looked at associations between five RA risk alleles and treatment response. *Methods*. We evaluated associations between anti-TNF treatment responses assessed by DAS28 change and by EULAR response at six months in 383 Portuguese patients. Univariate and multivariate linear and logistic regression analyses were performed. In a second step to confirm our findings, we pooled our population with 265 Spanish patients. *Results*. No association was found between *PTPRC* rs10919563 allele and anti-TNF treatment response, neither in Portuguese modeling for several clinical variables nor in the overall population combining Portuguese and Spanish patients. The minor allele for RA susceptibility, rs3761847 SNP in *TRAF1/C5* region, was associated with a poor response in linear and logistic univariate and multivariate regression analyses. No association was observed with the other allellic variants. Results were confirmed in the pooled analysis. *Conclusion*. This study did not replicate the association between *PTPRC* and the response to anti-TNF treatment in our Southern European population. We found that *TRAF1/C5* risk RA variants potentially influence anti-TNF treatment response.

## 1. Introduction

Rheumatoid arthritis (RA) is an inflammatory, chronic, and disabling disease. Methotrexate is the most widely used disease modifying antirheumatic drug (DMARD) in RA treatment. However, for refractory and severe cases, anti-tumor necrosis factor (anti-TNF) therapy has become a cornerstone of RA treatment strategy [1]. These drugs have revolutionized RA treatment and prognosis in the last 10–15 years. Nevertheless, only approximately one-third of patients achieve remission and the other third will eventually fail to respond [2]. In a multifactorial and polygenic disease like RA, it is expected that response to treatment may be influenced by genetic, clinical, and biological factors [3]. The identification of predictors of response is of crucial importance to optimize the cost-effective use of expensive medications, such as anti-TNF therapy. Large registries collecting information on sociodemographic characteristics, disease activity, functional status, and treatments have allowed the study of clinical predictors of response [4–7].

Genetic variants associated with RA susceptibility include the* HLA-DRB1* region containing shared epitope alleles (SE), which is also associated with severity [8, 9]. Outside the major histocompatibility complex (MHC),* PTPN22*,* TRAF1/C5*, and* TNFAIP3* loci were the most consistently associated with susceptibility and* TRAF1/C5* region also with RA severity [10, 11] and noncardiovascular mortality in some populations [12].

In the nineties, studies performed to look at associations between treatment response and the presence of SE indicated that the response to disease modifying antirheumatic drugs (DMARDs) such as methotrexate (MTX), in combination or monotherapy [13], and more recently with leflunomide [14], etanercept [15], and infliximab [16], was better in the presence of SE.

In recent years, several studies of potential associations between anti-TNF treatment response and polymorphisms in the promoter region of the* TNF* gene (positions −308 and −238), and other related genes such as* lymphotoxin-α* and* TNF receptors*, showed contradictory results [17–20]. With the increasing knowledge on RA pathophysiology and genomewide studies demonstrating that loci related with TNF signaling pathways such as the NF-kB signaling pathway (*TRAF1/C5*,* TNFAIP3*, and* REL*) and other pathological processes such as enhanced citrullination (*PADI4*) may increase RA risk, it is compelling to explore how those loci could also influence the anti-TNF response [21–28].

Cui et al., analyzing thirty-one risk allele variants, found that the major allele (G) of the rs10919563* PTPRC* locus, which is a known predictor of RA risk, was associated with an increased response to anti-TNF therapy, with stronger association in seropositive patients (either anticitrullinated peptides antibodies, ACPA, and/or rheumatoid factors (RF)) [29]. The authors did not find any association with treatment response among the other thirty RA-associated risk alleles studied. In that multicohort study, potential associations between response and* HLA-DRB1* were not assessed. One study from BRAGGSS, UK, showed no association between* HLA-DRB1* and* PTPN22* variants and response to anti-TNF treatment [30]. However, another study from the UK confirmed the association between* PTPRC* variants and response in the entire cohort, reporting no significance in the ACPA positive group alone [31].

The challenge over the next years will be to identify the RA stages in which genetic variants exert their maximum influence and also to unveil their clinical significance and usefulness as potential therapeutic targets or biomarkers [10].

In this study we aimed to replicate in a Southern European population the association between rs10919563* PTPRC* variants and the response to anti-TNF treatment found in previous studies. We also aimed to test whether* HLA-DRB1* and other five selected RA susceptibility genes may influence the response to anti-TNF treatment—that is, potential associations between anti-TNF treatment response and risk RA loci related with NF-kB signaling pathway (*TRAF1/C5*,* TNFAIP3, *and* REL*), citrullination (*PADI4*), and the genetic variants inside the MHC (*HLA-DRB1* ^*^ *04* high-resolution (4-digit) genotyping) and outside the MHC (*PTPN22* locus) with the strongest association with RA risk. The analyses were modeled adjusting for clinical variables that influenced treatment response.

## 2. Material and Methods

### 2.1. Patients

Primary analyses were performed upon Reuma.pt, the National Register for Rheumatic Diseases from the Portuguese Society of Rheumatology (SPR) established in 2008, which captures more than 90% of patients treated with biological therapies managed in rheumatology departments across Portugal [32]. The register is linked to the Biobanco-IMM [33]. Blood samples were collected from November 2010 up to May 2011 at six major centers. Information on disease activity and treatments has been collected by rheumatologists at every infusion for intravenous drugs and every 3 months for subcutaneous biologic therapies. The decision to initiate and maintain the treatment was guided by the SPR's recommendations [34]. RA patients fulfilling the American College of Rheumatology (ACR) 1987 revised criteria [35] were eligible for this study whether they were treated with an anti-TNF agent as the first biologic therapy, had a follow-up of at least six months, or had a blood sample collected for DNA assessment. Patients with self-reported non-Caucasian ancestry and those with missing values for DAS28 at baseline or at six months were excluded. Reuma.pt was approved by National Board of Data Protection and Health National Directorate.

In a second step for confirming our findings, we pooled our Portuguese sample with Spanish RA patients from the Rheumatology Department of Virgen de las Nieves (Granada, Spain) and Reina Sofia (Córdoba, Spain) Hospitals, selected with the criteria described above.

The study was conducted in accordance with the regulations governing clinical trials such as the Declaration of Helsinki and was approved by the Hospitals' Ethics Committees. Patients signed an informed consent for research use of their clinical data and blood samples.

### 2.2. DNA Extraction and Genotyping

DNA was extracted from whole blood using the QIAamp DNA Blood Mini Kit combined with the automated extraction device QIAcube (QIAGEN, Hilden, Germany), according to the manufacturer's instructions.

[*PTPRC*] rs10919563 (A/G) was previously reported in association with anti-TNF treatment response [29, 31]. The other five SNP markers were selected based on (1) relevance for RA biologic NF-kB pathway, [*6q23-TNFAIP3*] rs10499194 (C/T), [*REL*] rs13031237 (G/T), and [*TRAF1/C5*] rs3761847 (A/G); (2) role on citrullination process, [*PADI4*] rs2240340 (C/T); and (3) strong association with RA risk, [*PTPN22*] rs2476601 (A/G).* HLA-DRB1* ^*^ *04* high-resolution genotyping was performed by PCR sequence-specific primers (SSP) using Olerup SSP DRB1^*^04 typing Kit (Olerup SSP AB) as described in the manufacturers' protocols.

Samples were run with Luminex xMAP system (Tepnel Lifecodes). Allele call was obtained with Quicktype for Lifematch 2.6 software. The samples were genotyped using Taqman SNP genotyping assays (Applied Biosystems, Foster City, USA) as described in the manufacturers' protocols.

For purposes of quality control, 95% of sample threshold and 95% genotyping success threshold were used. Exclusion criteria also included a minor allele frequency <0.1 and deviation from Hardy-Weinberg equilibrium. In the end,* HLA-DRB1* ^*^ *04* and six allele variants were analyzed.

### 2.3. Statistical Analysis

#### 2.3.1. Outcome Measures and Covariates

Our primary outcome was the change in disease activity score in 28 joints including erythrocyte sedimentation rate measures (DAS28ESR) between the drug start date and six months of treatment (in our study, a positive variation means a decrease in disease activity at six months in comparison with baseline visit) [36]. Secondary outcome was the proportion of nonresponders versus good responders (excluding moderate responders) defined by the EULAR response criteria at six months [37]. Nonresponse was defined by an absolute change in DAS28 ≤ 0.6 or a change in DAS28 between 0.6 and 1.2 with a DAS28 at six months >5.1. Good response was defined as change in DAS28 of >1.2 and DAS28 at six months ≤3.2. In this study, nonresponse was the reference category, with logistic regression analysis modeling the probability of achieving good response. For both outcomes, covariates' coefficient > 0 or odds ratio (OR) > 1 predicted favorable response.

The predictors of interest were the minor allele variants of* PTPRC* and the five SNPs described above as well as the presence of SE (*DRB1* ^*^ *0401/04/05/08* and* DRB1* ^*^ *1001*), as previously described in the RA Portuguese population [8].

Covariates collected at drug start date (baseline visit) were gender, age, age at diagnosis, disease duration, years of education, smoking (ever/never), RF (positive/negative), ACPA (CCP2, positive/negative), extra-articular manifestations (yes/no), concomitant therapy with corticosteroids (yes/no), any disease modifying antirheumatic drugs (DMARDs) including MTX (yes/no), DAS28, Health Assessment Questionnaire (HAQ), and physician's global assessment of disease activity (PhGA). We tested the association between these variables and change in DAS28 at six months by univariate analyses. Then, we built a multivariate linear model with the significant baseline clinical covariates (at a *P* < 0.05). The variables that remained significant after adjustment entered the multivariate model for each SNP.

In a second step, in order to confirm our results, we pooled the Spanish population and looked at associations between the SNPs and treatment response.

#### 2.3.2. Primary Analysis

Each SNP was tested for an association with the anti-TNF response, taking anti-TNF drugs as a group. Univariate linear regression analyses for the primary outcome and logistic regression for the secondary outcome were performed, using additive models. Homozygotes for major alleles were classified as 0, 1 for heterozygotes, and 2 for minor allele homozygotes. The response was also modeled with multivariate models including significant baseline clinical predictors for treatment response.

#### 2.3.3. Secondary Analyses

For the loci that presented a significant association with treatment response in the primary analysis, we tested the same relationship by stratifying the patients in ACPA positive and ACPA negative groups.

The study had >80% power to detect a change of >0.6 in the DAS28 score (considered a clinically meaningful change [37]) for allele frequencies > 0.1 and a type I error of <0.05.

There were no assumptions about the direction of effect on treatment response. Results were considered significant for a two sided *P* value <0.05. Analyses were performed using SAS version 9.3 (SAS Institute, Cary, NC, USA).

## 3. Results

*PTPRC*, additional five SNP markers, and* HLA-DRB1* genotyping were assessed in 416 Portuguese patients. Twenty-seven reported a non-Caucasian ancestry and were excluded. Six were excluded for absence of DAS28 at baseline or at six months, leaving 383 patients for analysis.  Table 1 describes the baseline demographic and clinical characteristics for the 383 RA Portuguese patients included in the study.

At six months, 119 (31.1%) patients were classified as good responders, 175 (45.7%) as moderate responders, and 89 (23.2%) as nonresponders according to the EULAR response criteria [37].

Number of years completed at school, HAQ, and DAS28 at baseline were found to have a significant association with treatment response at six months and were included in the multivariate models (Table 2).

We did not find association between* PTPRC* rs10919563 and anti-TNF treatment response (Table 3).

Univariate analysis looking for association between the other five risk alleles and anti-TNF treatment response demonstrated a worse response for minor (G) allele, rs3761847 SNP, in* TRAF1/C5* region either measured by a change in DAS28 at six months (coefficient (coef.) −0.24; 95% confidence interval (CI) −0.43, −0.06; *P* value of 0.009) or by the proportion of good responders versus nonresponders at six months (OR 0.61; CI 0.41, 0.92; *P* value of 0.018). After adjusting for years of school completed, HAQ, and DAS28 at baseline, in two multivariate models, this association remained significant for a *P* value < 0.05 (Table 4). In the univariate linear model, rs3761847 accounted for 1.75% of the change in DAS28 at six months. The multivariate model with the clinical covariates described above explained 23.9% of this change. When rs3761847 was added to the clinical model, the *R* ^2^ increased to 25.4%.

There were no significant relationships between the other SNP markers tested and response to therapy (Table 3). Similarly, SE was not associated with response to anti-TNF therapy in our population (coef. 0.21, *P* value 0.21; OR 1.24, CI 0.56, 2.71).

In a secondary stratified analysis, the relationship between rs3761847 and treatment response in 278 ACPA positive and 105 ACPA negative patients was tested, but no association was detected (ACPA positive group, coef. −0.16, *P* value 0.09 and ACPA negative group, coef. −0.41, *P* value 0.04).

We replicated these findings pooling 265 Spanish RA patients and testing the association between the risk alleles and anti-TNF treatment response (results not shown).

## 4. Discussion

Our study did not replicate the association previously published between* PTPRC* rs10919563 variant and the response to anti-TNF therapy in patients with RA. The analyses suggest that in our Southern European population, the minor (G) allele rs3761847 in the* TRAF1/C5 *locus might have an association with poor response to anti-TNF treatment at six months. These results were consistent using either the absolute change in DAS28 or the proportion of good/non-responders as outcomes in univariate and multivariate models adjusted for clinical predictors of response. The other four RA susceptibility loci tested and the* HLADRB1 *were not associated with anti-TNF response.

In 2007,* TRAF1/C5* was identified as a risk locus for RA by Plenge and colleagues in a GWAS [21] and by Kurreeman et al. in a candidate gene approach [38]. More recently, it was shown to be a marker of disease severity [10, 11] and in one study of noncardiovascular mortality [12]. No previous studies have reported the association with response to anti-TNF therapy. Due to a high level of linkage disequilibrium between the genes encoding TNF receptor associated factor 1 and complement component 5, it is currently not possible to assure which of these two genes at 9q33.2 encloses the causal variant. Both are possible candidates. The protein, TNF receptor-associated factor 1 (TRAF1), is a member of the TNF receptor (TNFR) associated factor (TRAF) protein family and is encoded by the* TRAF* gene. TRAF proteins associate with and mediate the signal transduction from various receptors of the TNFR superfamily. TRAF1 and TRAF2 form a heterodimeric complex, which is required for TNF mediated activation of MAPK8/JNK and NF-kB. The protein complex interacts with inhibitor-of-apoptosis proteins and mediates the antiapoptotic signals from TNF receptors. TRAF 1 is a negative regulator of TNF receptor and Toll-like receptor signaling and may contribute to the proliferation of T cells. rs3761847 is located at the upstream of* TRAF1* and the downstream of the complement fraction* C5 *[39]. The clinical and biologic data for* C5 *are equally relevant. The complement pathway has been implicated in the pathogenesis of rheumatoid arthritis for more than 30 years. C5 cleavage generates the proinflammatory anaphylatoxin C5a, as well as C5b, which initiates the generation of the membrane-attack complex. C5-deficient mice are resistant to inflammatory arthritis in models with a dominant humoral component [21, 40]. It is compelling to hypothesize that this variant or other causative variants at this locus may influence the function or expression levels of TRAF1 and/or C5, affecting RA susceptibility, severity, and anti-TNF treatment response. Functional studies are warranted to confirm these hypotheses. Nevertheless, our result could be that false positive and studies with other populations are required to confirm the replication of these findings.

We did not find any significant associations between response to treatment and the presence of SE, neither with the other four RA risk allele variants related with NF-kB signaling pathway nor with citrullination, chosen for its high association with RA susceptibility. We were also not able to replicate the previously reported* PTPRC* association with treatment response in our Southern European population. Plant et al. reported a *P* value of 0.04 for* PTPRC* association with anti-TNF treatment response and no significance for the ACPA positive patients in the stratified analysis. This result was strengthened with a meta-analysis combining their data with the Cui et al. study [29]. Although our sample size was large enough to detect allele variants association with response with a power > 80% for minor allele frequency (MAF) > 0.1,* PTPRC* showed a MAF of 0.11 which might have made difficult the detection of association. The different genetic background of our population could also account for the lack of replication. In a recent study, we and two Japanese groups also failed to replicate the results found in Northern European populations [41].

We are far from understanding the genetic mechanisms that underlie treatment response in patients with RA, as demonstrated by the large proportion of the variance explained by clinical factors compared to that explained by a single SNP. The ultimate goal for genetic, laboratory, and clinical predictors of treatment response studies is personalizing treatment and medicine practice by identifying biomarkers and specific phenotypes clinically useful for improving the therapeutic strategy. The identification of individual predictors may contribute to building complex algorithms aimed at improving the prediction of a better/worse response to anti-TNF drugs and other classes of biologic therapies.

## 5. Conclusions

We were not able to replicate the previously reported* PTPRC* rs10919563 association with treatment response in our Southern European RA population.

The minor (G) allele of rs3761847 in the* TRAF1/C5 *locus, which is a susceptibility factor for RA related to TNF signaling, was associated with a poor response to anti-TNF treatment at six months, using either the absolute change in DAS28 or the proportion of good responders and nonresponders as outcomes. This association was also observed after adjustment in a multivariate model with baseline clinical predictors of response.

We did not find any significant associations between response to treatment and the presence of SE, neither with the other four RA risk allele variants related with NF-kB signaling pathway nor with citrullination, chosen for its high association with susceptibility.

Additional studies in other populations are necessary to confirm the relevance of these findings.

## Figures and Tables

**Table 1:** Baseline demographic and clinical characteristics of the 383 rheumatoid arthritis patients treated with anti-TNF drugs.

TNF inhibitor	
Adalimumab	79 (20.6)
Etanercept	139 (36.3)
Golimumab	10 (2.6)
Infliximab	155 (40.5)
Age (years)	52.5 (12.2)
Disease duration (years)	10.7 (8.9)
Female	343 (89.5)
Rheumatoid factor	290 (75.7)
ACPA	278 (72.6)
Extra-articular manifestations	91 (23.7)
Smoking-ever (*n* = 367)	71 (19.3)
Education (years)	7.1 (4.6)
DMARDs (*n* = 377)	346 (91.8)
MTX (*n* = 377)	310 (82.2)
Corticosteroids (*n* = 377)	277 (73.5)
DAS28 ESR	5.77 (1.1)
Physician global assessment (mm)	56.1 (16.5)
Health Assessment Questionnaire	1.45 (0.58)

Values shown are means (SD) or *n* (%).

TNF: tumor necrosis factor; ACPA: anticitrullinated peptides antibodies; DMARDs: disease modifying antirheumatic drugs; MTX: methotrexate; DAS: disease activity score; ESR: erythrocyte sedimentation rate.

**Table 2:** Multivariate model of baseline demographic and clinical variables as predictors of response to anti-TNF treatment at 6 months.

Baseline variables	Coefficient (*P* value)
Age (years)	−0.01 (0.25)
Disease duration (years)	0.01 (0.24)
Female gender	−0.01 (0.95)
ACPA (positive)	−0.07 (0.64)
Smoking (ever)	−0.06 (0.72)
Higher education (years)	0.03 (0.03)^*^
Corticosteroids (yes)	−0.16 (0.26)
DMARDs (yes)	0.05 (0.83)
Extra-artic. manif. (yes)	0.10 (0.49)
HAQ	−0.40 (0.002)^*^
PhGA (mm)	−0.001 (0.68)
DAS	0.60 (<0.001)^*^

^*^ *P* value <0.05.

The probability of response to anti-tumor necrosis factor therapy at 6 months was modeled in a multivariate linear regression analysis.

The change in disease activity score assessing 28 joints between the baseline visit and the visit after 6 months of therapy was the continuous outcome.

ACPA: anticitrullinated peptides antibodies; DMARDs: disease modifying antirheumatic drugs; Extra-artic. manif.: extra-articular manifestations; HAQ: Health Assessment Questionnaire; PhGA: physician global assessment; DAS: disease activity score.

**Table 3:** Minor allele genetic variants as predictors of response, analyzed by univariate and multivariate linear and logistic regression models.

SNP	Gene	Chr Position (bp)	Minor allele	MAF	Absolute change in DAS		EULAR good response versus nonresponse	
					*n* = 383		*n* = 208 (good responders = 119, nonresponders = 89)	
					Univariate additive linear model	Multivariate additive linear model	Univariate additive logistic model	Multivariate additive logistic model
					Coef (95% CI)	Coef (95% CI)	OR (95% CI)	OR (95% CI)
					*P* value	*P* value	*P* value	*P* value
rs2476601	*PTPN22 *	1	A	0.11	0.28 (−0.01, 0.58)	0.12 (−0.15, 0.40)	1.68 (0.88, 3.21)	1.71 (0.82, 3.54)
		114,179,091			0.058	0.38	0.12	0.15

rs2240340	*PADI4 *	1	T	0.46	−0.11 (−0.29, 0.07)	−0.09 (−0.25, 0.08)	0.79 (0.54, 1.16)	0.81 (0.52, 1.24)
		17,507,279			0.22	0.30	0.22	0.33

rs13031237	*REL *	2	T	0.45	−0.09 (−0.26, 0.09)	−0.14 (−0.31, 0.02)	0.91 (0.62, 1.33)	0.77 (0.50, 1.19)
		60,989,633			0.34	0.09	0.62	0.24

rs10499194	*TNFAIP3 *	6	T	0.29	−0.05 (−0.26, 0.15)	−0.06 (−0.26, 0.12)	0.79 (0.50, 1.25)	0.86 (0.52, 1.40)
		138,002,637			0.60	0.50	0.31	0.55

rs10919563	*PTPRC *	1	A	0.11	−0.05 (−0.34, 0.23)	0.05 (−0.21, 0.32)	1.21 (0.65, 2.22)	1.61 (0.79, 3.26)
		196,967,065			0.72	0.69	0.55	0.19

Values were significant for *P* < 0.05.

The analyses of response were modeled for the minor alleles. Additive models were used taking the homozygote for the major allele as the reference variable.

383 patients were included in the primary analysis (the outcome was the absolute change in the disease activity score (DAS28) between the baseline and the 6-month visit).

208 patients were assessed in the secondary analyses; 119 were good responders and 89 nonresponders according to the EULAR response criteria.

Values were presented as regression coefficient (coef.), 95% confidence intervals (CI), and *P* values (*P*) for linear regression analyses and as odds ratio (OR), 95% confidence intervals (CI), and *P* values (*P*) for logistic regression analyses.

In multivariate models, covariates included were number of years completed at school, health assessment questionnaire, and disease activity score at baseline.

SNP: single nucleotide polymorphism; Chr: chromosome; MAF: minor allele frequency.

**Table 4:** Association of the rs3761847 single nucleotide polymorphism of *TRAF1/C5* locus with the response to anti-TNF treatment.

SNP	Ch	Position Bp	Genotype	Count	MAF	Change in DAS	Absolute change in DAS		EULAR good response versus nonresponse	
							*n* = 383		*n* = 208 (good = 119, non = 89)	
							Linear regression models		Logistic regression models	
							Univariate	Multivariate	Univariate	Multivariate
rs3761847	9	122,730,060	11	167		1.95 (1.26)	Coef. −0.24	Coef. −0.23	OR 0.61	OR 0.58
			12	165	0.35	1.82 (1.31)	CI −0.43, −0.06	CI −0.40, −0.06	CI 0.41, 0.92	CI 0.37, 0.91
			22	51		1.38 (1.19)	*P* 0.009	*P* 0.009	*P* 0.018	*P* 0.019

Values were significant for *P* < 0.05.

The analyses of response were modeled for the minor (G) allele, which is the risk allele for RA. Additive models were used with the homozygote for the two major alleles as the reference variable.

383 patients were included in the primary analysis (the outcome was the absolute change in the disease activity score (DAS28) between the baseline and the 6 month visit).

208 patients were assessed in the secondary analyses, 119 were good responders and 89 non-responders according to the EULAR response criteria.

Values were presented as regression coefficient (coef.), 95% confidence intervals (CI) and *P*-values (*P*) for linear regression analyses and as odds ratio (OR), 95% confidence intervals (CI) and *P*-values (*P*) for logistic regression analyses.

In multivariate models covariates included were number of years completed at school, health assessment questionnaire (HAQ) and disease activity score at baseline.

SNP: single nucleotide polymorphism; Ch: chromosome; Genotype 1 = major allele, 2 = minor allele; MAF: minor allele frequency; DAS: disease activity score.
